# Case report: Injury plausibility for skull fractures in two kittens

**DOI:** 10.3389/fvets.2026.1751730

**Published:** 2026-06-25

**Authors:** Jennifer A. Woolf

**Affiliations:** Woolf Veterinary Forensics Consulting, Inc., Dublin, CA, United States

**Keywords:** animal cruelty, blunt head injuries, skull fractures, veterinary forensic postmortem examination, veterinary forensics

## Abstract

A forensic postmortem examination (FPME) was performed on two kittens. In each case, investigators were given conflicting stories describing how the kitten died. The goal for each FPME was to determine the cause of death with the hope of also determining which story was more plausible. In both cases, animal control officers responded to a call to retrieve a deceased kitten. In the first case, the kitten’s owner reported the kitten had died when thrown to the floor by the owner’s boyfriend; the boyfriend reported he had accidentally dropped the kitten. In the second case, an ex-girlfriend of the owner reported the owner had crushed the kitten’s head against a wall; the owner stated the kitten had come between a wall and a piece of furniture being moved, causing its accidental death. Findings from the radiographs, FPME, and histopathology showed the cause of death in the first case to be blunt force trauma resulting in a non-pathological skull fracture and traumatic brain injury (TBI). The distribution and type of injuries were consistent with being thrown against the floor and were not consistent with an accidental fall. Findings from the radiographs and FPME of the second case also showed blunt force trauma to the head leading to TBI and death, but could not determine which story was more plausible due to a limited investigation resulting in a lack of information necessary to make the distinction. Together, the two cases demonstrate the importance of a FPME in cases of suspicious death while also demonstrating the limitations of FPME alone in determining whether a death is accidental or malicious. They also demonstrate the limitations of current literature on animal abuse and the need for more studies and case reports specific to animal maltreatment.

## 1 Introduction

Two cases of kittens with fatal skull fractures due to potential animal cruelty are presented. In the first, the owner’s boyfriend claimed that the kitten died accidentally when it was dropped, but the owner said the boyfriend intentionally threw it to the floor. In the second case, the owner’s ex-girlfriend reported that the owner crushed the kitten’s head against a wall, but he said the kitten accidentally became caught between a piece of furniture that was being moved and the wall.

One of the challenges of investigating animal cruelty is determining the true cause of injury when there are conflicting stories between the alleged perpetrator and witness(es), such as the two cases here. Often, one person’s story indicates an accidental injury or death while another person’s story indicates animal maltreatment. Those investigating the case must then use available evidence —including, but not limited to, the forensic postmortem examination (FPME) — to determine which story is the most plausible to explain the injury or death. By reasoning through what is known in the literature about injuries to animals, knowledge of normal animal behavior, and knowledge of the biomechanics of injury, veterinarians may be able to determine whether a given story is consistent with the animal’s injuries.

There is more extensive information in the literature on child abuse than on animal abuse. In a study of child abuse by Pierce et al. (1) it was noted that different characteristics in cases of femoral fractures from stair falls in children could help determine if a case was more likely due to abuse or accident. This was termed injury plausibility. These characteristics included histories that were vague on details, fractures that did not have biomechanical conditions agreeing with the history provided, delays in seeking care despite the child being in obvious pain, and additional injuries found during the initial examination.

Similar to what is seen in child abuse (1, 2), certain false histories such as an accidental fall are not uncommon in animal cruelty. A story that is inconsistent with the injuries seen is one potential red flag for abuse, whether in animals or children. While the FPME is important to any given case, there are often multiple possible explanations for the findings. Stories that are vague or incomplete may require further investigation from professionals in other fields such as animal control and law enforcement before a determination of intentional abuse or accident can be made.

In child abuse studies looking at falls of less than 3 feet in infants, severe head injury is uncommon (3), despite the increased mass of an infant’s head in relation to the body. No studies were found by the author that examined the rate and type of injury in cats that fall short distances. Most of what is known about fall-related injuries in cats are from studies of falls from significant heights as seen in high-rise syndrome (HRS) where cats fall a distance of at least 2 stories (approximately 7.3 m). In HRS, injuries to the head specifically tend to involve orofacial injuries, and both traumatic brain injury (TBI) and death are uncommon (4–6). These studies in children and cats are important because they suggest that accidental falls in the home such as the alleged drop in the first case are unlikely to result in serious harm or fatal injury.

Both kittens in the cases presented had skull fractures. A study by Knight and Meeson (7) looked at skull fractures in cats that had traumatic head injuries. The trauma was classified as either falling from an unspecified height, road traffic accident (RTA), bite wound, or gunshot wound. Of the 75 cats in the study, 89% were injured by RTA and only 3% of the skull fractures were caused by falls. Approximately 70% of all of the cats had fractures of the upper and lower jaws and craniofacial areas. Only 7 (9%) of the cats had caudal skull fractures, defined as those involving the calvarium, parietal, occipital, presphenoid and basisphenoid bones. Of those 7 cats, none of the fractures were associated with falls. Knowing the typical pattern of skull fractures due to trauma can help discern inconsistencies in stories provided to explain an animal’s injuries such as those of the two kittens.

In all of these studies examining traumatic injuries in cats (4–7), the indicated cause of injury is something other than abuse; it is unknown whether the history provided for the cause of injury or death was plausible because it is unknown if the possibility of animal cruelty was ever considered. For instance, in the Knight and Meeson study (7), RTA included cats witnessed as having been hit by a car and those that were found by a road. It is possible some of those cats were mis-classified as RTA when they were actually due to another type of trauma, including the possibility of abuse. Nevertheless, these studies provide important information for comparison of the injuries sustained by the two kittens in this case report to what is known about falls and traumatic skull fractures in cats.

Another inconsistency sometimes seen in cases of animal maltreatment is a description of the animal’s behavior that does not seem likely given what is known of normal behavior for the species. For instance, cats naturally have an instinct to right themselves when falling so they will land on their feet; this is called the righting reflex. In a study by Sechzer et al. (8), kittens aged 0–70 days were tested three times per week for their postural reflexes until the reflex was fully developed. To test for the righting reflex, kittens were held in a supine position then dropped a distance of about one foot (approximately 61 cm). With this test, the researchers found the righting reflex begins development at 3 weeks of age and is fully developed by 6 weeks of age. They also demonstrated that a cat can right itself quickly, needing as little as a foot of distance to do so. This information about falls, skull fractures, and the righting reflex can be used to determine the plausibility of the injuries to the two kittens.

## 2 Case description

In case number one, animal control services were requested to dispose of a deceased kitten. The animal control officer (ACO) was told by the owner that her boyfriend (height 1.9 m) had thrown the kitten against the hardwood floor from a standing position. Following impact the kitten died less than a minute later. When the ACO interviewed the boyfriend, he said that he had dropped it accidentally. A FPME was performed to determine the cause of death and the plausibility of each story.

**Figure 1:**
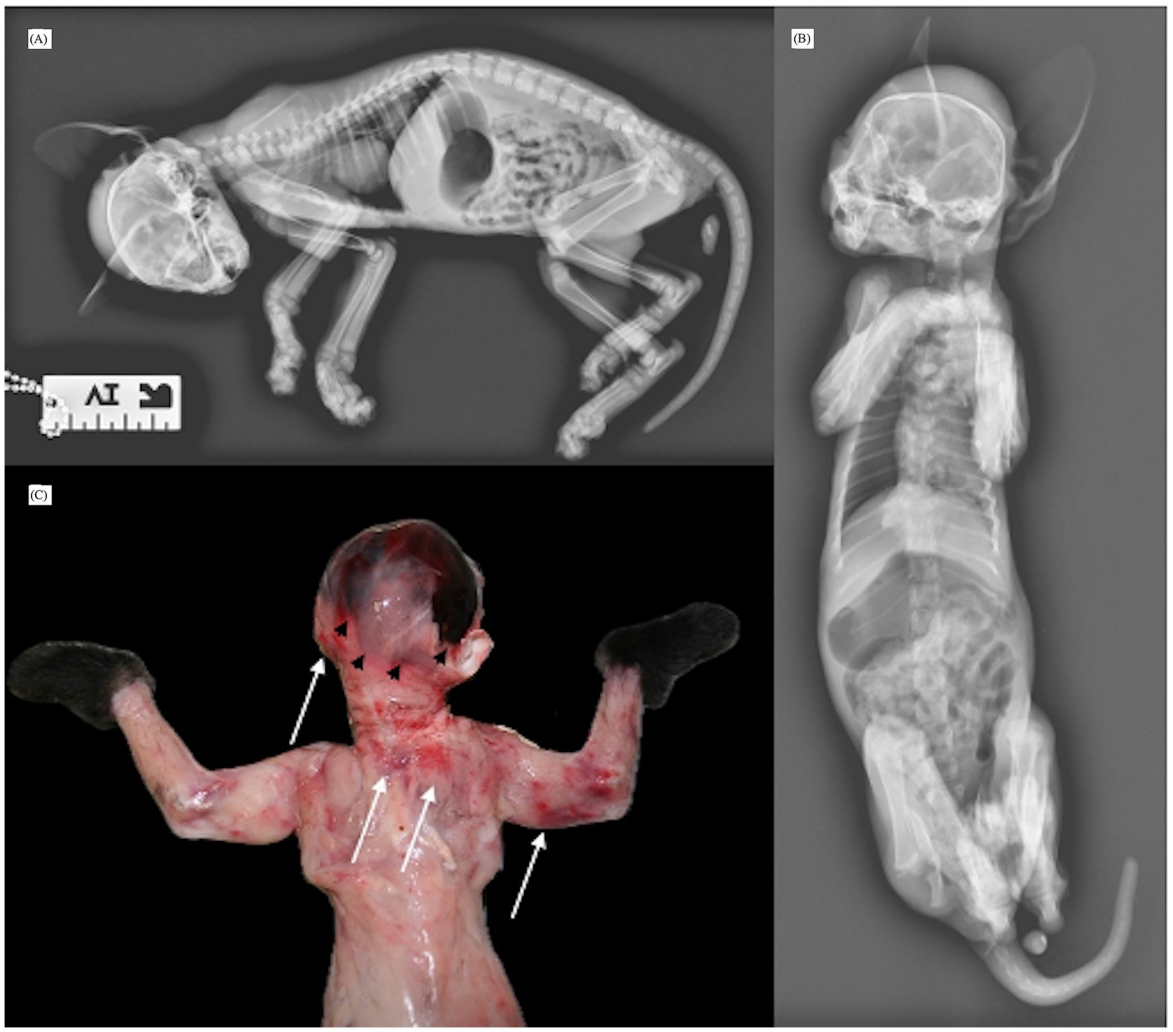
Case 1 – **(A)** Postmortem radiograph in approximately right lateral positioning. Swelling can be seen in the soft tissues at the dorsocaudal aspect of the head. **(B)** Postmortem radiograph in approximately ventrodorsal positioning. Swelling is seen at the dorsal aspect of the head. **(C)** Dorsal aspect of head, neck, shoulders, and thorax, and lateral aspects of forelimbs of the kitten as seen at the forensic postmortem examination. Note the petechiae and ecchymoses over the shoulders and humeri, more concentrated on the right than the left (white arrows) and the hemorrhage into the muscles and soft tissues over the caudal aspect of the head (black arrowheads).

Prior to the FPME, full body, postmortem radiographs were obtained [right lateral (Figure 1A) and ventrodorsal (Figure 1B)]. The images showed soft tissue swelling dorsal to the cranium without foreign material or gas. No fractures were identified. There was also concern for generalized decreased bone density.

On FPME, the 6-week-old, intact female, domestic shorthair kitten was thin, had fleas, and a small amount of formed feces attached to the perianal fur. The dorsal aspect of the head was soft on palpation. Diffuse scleral hemorrhage was noted in the dorsolateral aspect of the right eye. There were no fractured teeth, no oral injuries, and no fluid in the ear canals or nostrils.

**Figure 2:**
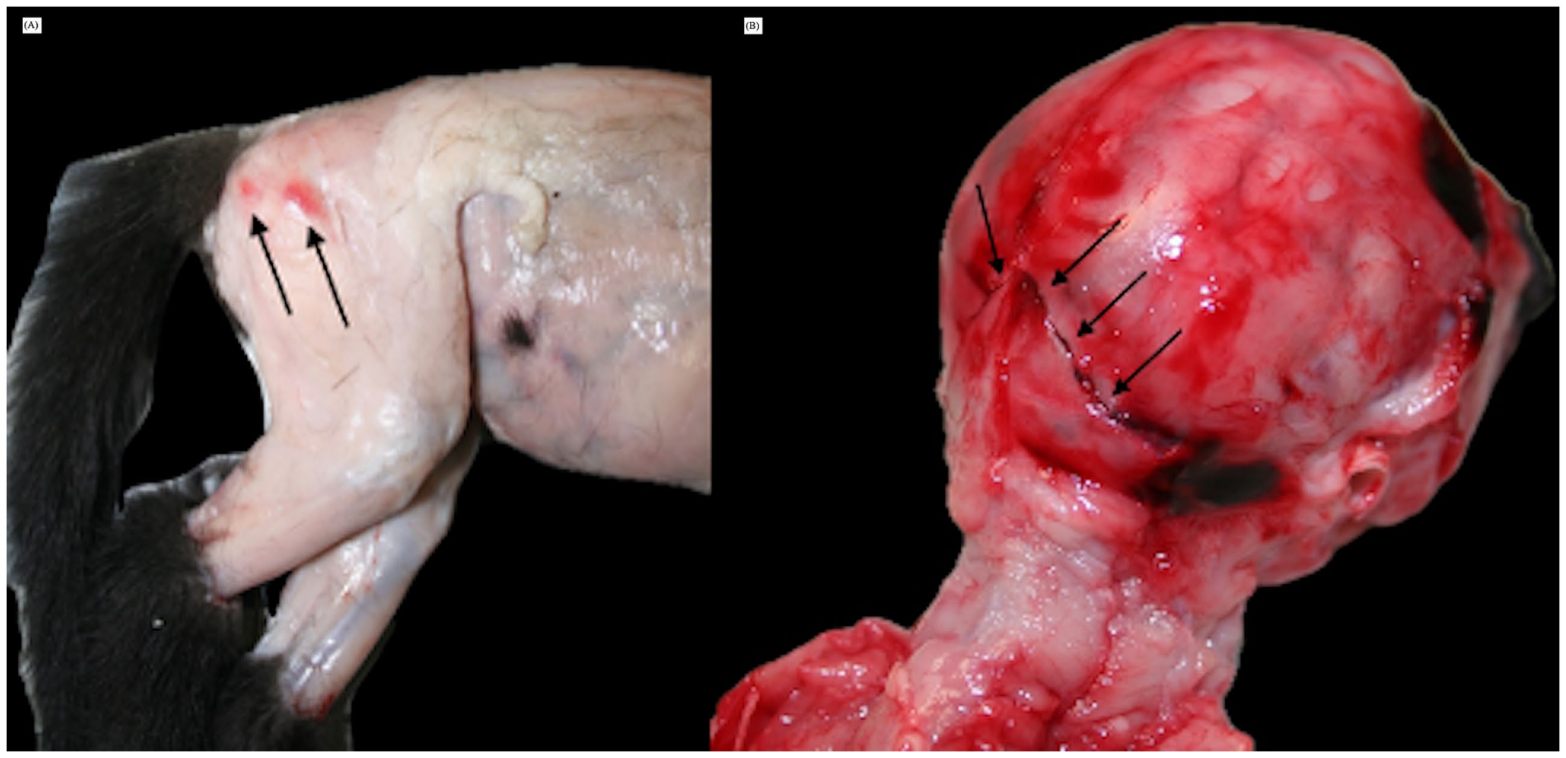
Case 1 – **(A)** Right lateral aspect of pelvic region of the kitten as seen at the forensic postmortem examination. Note the ecchymoses over the right hip (arrows). **(B)** The caudal and right lateral aspect of the skull of the kitten as seen at the forensic postmortem examination. The fracture follows the normal suture lines between the occipital and parietal bones (arrows).

Reflection of the skin over the head revealed mottled hemorrhagic, edematous subcutaneous tissues at the dorsal aspect extending from the level of the eyes caudally to the atlanto-occipital junction, right laterally to the ear, and left laterally to the level of the eye. Multifocal and coalescing petechiae and ecchymoses were noted on the subcutis over the dorsal aspect of the neck and shoulder region extending laterally to both elbows, more extensive on the right than the left (Figure 1C). Ecchymoses were also noted on the subcutis of the right hip (Figure 2A).

Internal FPME showed multifocal and coalescing petechiae and ecchymoses on the pleural aspect of the right, mid-dorsal thoracic wall at ribs 1–5 and on the diaphragmatic aspect of the right side of the liver.

The muscles of the head were removed revealing a single, complete, non-displaced fracture at the occipital bones extending from one side of the skull to the other in an inverted, asymmetrical “V,” longer on the right side. This fracture followed the normal suture lines between the occipital and parietal bones (Figure 2B). Within the cranial vault, hemorrhage was noted in the surrounding tissues of the brain and meninges.

On histopathology, the occipital lobes and cerebellum had acute hemorrhage into the leptomeninges. Sections of the occipital and parietal bones had acute hemorrhage next to the suture lines where the fracture was located. The bones had normal architecture with no evidence of disease or a congenital or genetic disorder. Sections from a femur were examined and also found to be normal negating the radiographic concerns for decreased bone density.

The cause of death was TBI secondary to the skull fracture, consistent with being forcefully picked up over the shoulders and thrown to the ground; it was inconsistent with an accidental drop.

In the second case, the owner’s ex-girlfriend reported that the owner told her he had killed his kitten by crushing the kitten’s head against a wall; she had not witnessed this personally. The owner said it died after becoming caught between the wall and a piece of furniture that was being moved.

**Figure 3:**
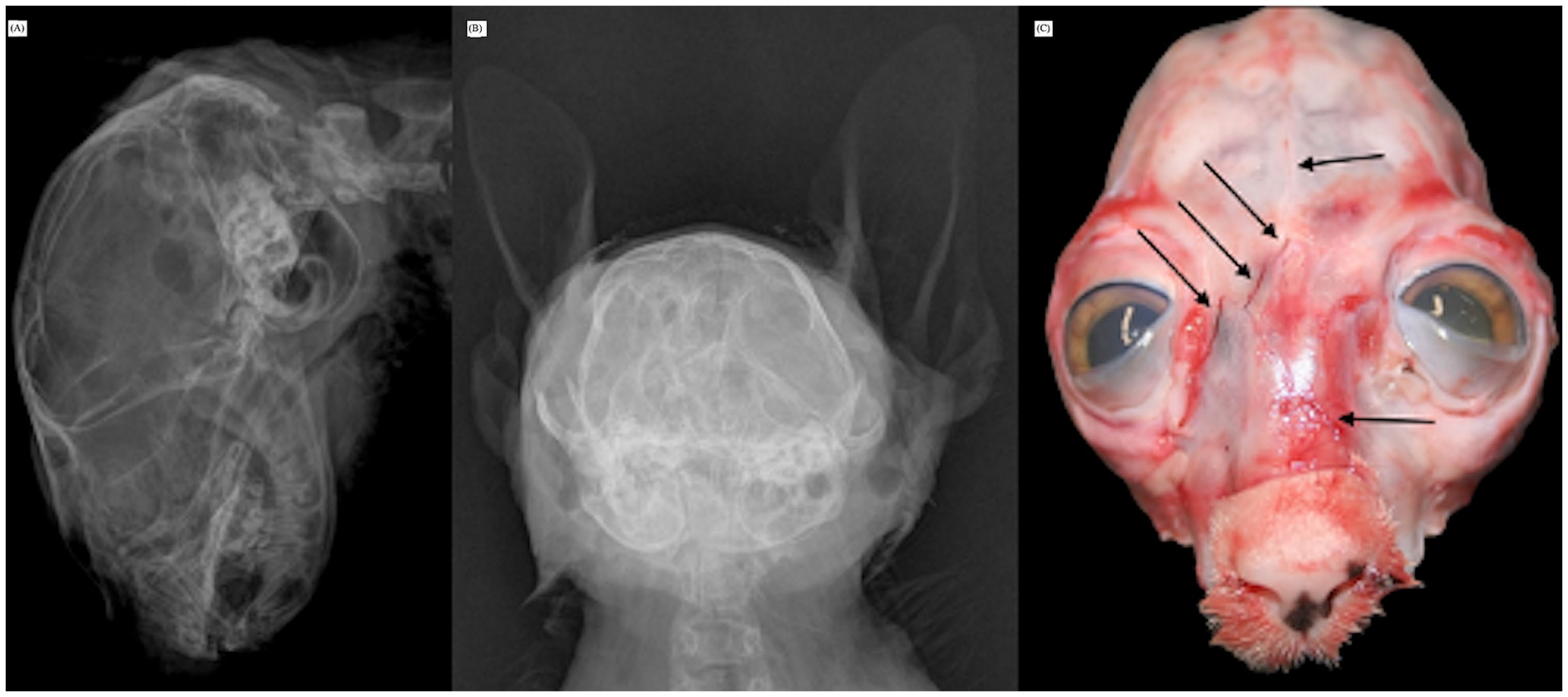
Case 2 – Postmortem radiographs of the skull in **(A)** approximately right lateral and **(B)** approximately rostrocaudal positioning. The author was concerned for one or more skull fractures based on these images but could not determine with certainty that fractures were present versus that positioning artifacts were being seen. **(C)** The craniodorsal aspect of the skull of the kitten as seen at the forensic postmortem examination. Note the multiple fractures in the nasal, frontal, and maxilla bones, some of which follow the natural suture lines (arrows).

Right lateral and ventrodorsal postmortem radiographs were taken of the entire body. Findings included a suspicion for one or more skull fractures (Figures 3A,B). No other significant findings were noted.

A FPME was performed on an approximately 10-week-old, intact male, domestic shorthair kitten. The kitten’s body was in good condition with no indication of external parasites. All of the deciduous teeth and claws were intact. No hemorrhage was associated with the globe of either eye, but the eyelids were bruised. Blood-tinged fluid was on the face, nose, and front paws. Additionally blood was noted within the right ear canal and at both nares. Clotted blood was found within the mouth, nasal cavities, trachea, and esophagus.

Reflecting the skin over the head revealed moderate edema and contusions on the left caudal aspect of the head with corresponding hemorrhage on the subcutis of the overlying skin. The contusions extended through the muscles and the soft tissues to the skull. No other contusions were noted anywhere else on the body or the subcutis. Multifocal petechiae and ecchymoses were present on approximately 10% of the lungs with no other significant findings within the thoracic or abdominal cavities or viscera.

After removal of soft tissue, multiple fractures were identified in the skull. Most fractures followed suture lines and affected the nasal, frontal, maxilla, temporal, parietal, basisphenoid, and occipital bones (Figure 3C). The mandibles were intact and without significant findings. Hemorrhage was seen within the skull and on the brain, particularly around and between the caudal cerebrum and the cerebellum. Histopathology was not performed for financial reasons.

The cause of death for this second kitten was also determined to be TBI secondary to multiple skull fractures. However, because the forces involved in the two stories were potentially quite similar (a crushing force applied to both sides of the kitten’s head), the plausibility of accident versus abuse could not be made.

## 3 Discussion

In both cases, the main goals of the FPME were to determine the cause of death and whose story better fit the injuries seen. Cause of death was clearly established in both to be TBI secondary to skull fractures. Determining the plausibility of each story, however, was only possible in the first case.

The velocity of a thrown kitten can significantly exceed the velocity reached by a dropped kitten. This will increase the force of impact and subsequently increase the amount of expected injury compared to an accidental fall (9). The impacted surface will also affect the severity of injury. Greater injury is likely when a cat impacts hard surfaces such as hardwood floors compared to soft surfaces such as grass (10). Also, the increased velocity from being thrown may not allow adequate time for a kitten to right itself, which can change the location of expected injury relative to a kitten falling accidentally. A kitten that rights itself may have injury on the ventral aspects of the body if it sustains any injury at all. One that is unable to right itself before impact is more likely to sustain injury to the dorsal and/or lateral aspects of the body.

Therefore, it was determined that the injuries to the first kitten were consistent with being picked up forcefully over the shoulders and then thrown to the ground. This conclusion was based on multiple factors. One, had the kitten been dropped accidentally, it would have righted itself to land on its feet, not its head. Two, the contusions over the dorsal neck and shoulders were consistent with being grabbed forcefully before being hurled to the floor. Three, the contusions were slightly more prevalent on the right side of the body than the left, consistent with impacting the floor in a dorsally recumbent and slightly right-lateral position. Four, cats that suffer trauma do not typically have fractures at the caudal aspect of the skull (7). Five, had the injuries occurred due to an accidental drop, it is more likely they would have involved the ventral regions. Finally, due to the relatively minimal force that would come from being dropped when compared to being thrown, serious injury from being dropped accidentally was simply unlikely without an underlying condition predisposing the kitten to injury.

The second kitten’s injuries were isolated to the head and could have occurred if the kitten had its head crushed against a solid surface by a significant force without having any other part of the body hit that surface with an equivalent force. This could be consistent with the story of the owner intentionally crushing the kitten’s head against a wall. However, there was not enough information known about the story of alleged movement of furniture to determine it implausible. Other information that would have been useful would have included the weight, dimensions, and style of furniture (e.g., sofa vs. dresser, flat on the floor vs. on feet), the manner in which it was being moved (e.g., carried vs. slid across the floor), the surface on which it was being moved (e.g., carpet vs. hardwood), and the speed at which the owner could move it. It would also need to be known how much of the kitten the owner claimed was caught between the furniture and the wall. Without this additional information, it could not be determined whether the ex-girlfriend’s story or the owner’s story was more plausible nor could either story be dismissed.

An important limitation in many investigations of animal maltreatment is the inability to know with absolute certainty what caused the injury. There may never be a confession to the alleged crime and the evidence may not be adequate to remove all doubt about the cause. While the evidence was enough to determine which story was most plausible for the injuries in the first kitten, it was not sufficient to differentiate between the alleged stories explaining the injuries in the second kitten.

Postmortem radiographs are an essential part of a FPME but they also have limitations. Because they are taken after death, the body may be in rigor mortis or frozen making standard positioning impossible. This issue was seen with both kittens. Additionally, the images are often taken while the body is still bagged. This not only adds to difficulty in positioning but it can also add artifact. Decomposition may cause artifact as well, such as increased intestinal gas which may not have been present antemortem. Nevertheless, postmortem radiographs allow the veterinarian to look for fractures – whether acute, healing, or healed – and foreign bodies such as projectiles. These can easily be missed on a FPME unless the veterinarian is expecting them in advance. Computed tomography (CT) can be more useful than radiographs, but often this is either inaccessible or unaffordable for an agency.

Another limitation in investigation of animal maltreatment is a lack of published studies on the injuries seen in these cases. It would be unethical to perform prospective studies in live animals to determine the type of injuries seen with different types of abuse. Studies in deceased animals may not be helpful because of the lack of a vital reaction normally seen with antemortem injuries. Additionally, deceased animals will not stand, move, or react in the way live animals do making it difficult to replicate the dynamics involved in abuse situations. Retrospective studies depend on a case having been generated by a veterinarian, animal control, or law enforcement. Because many instances of animal abuse go unreported, they will not be available for study.

Finally, studies already published involving trauma to animals may unknowingly include false stories of accidental injury. Rarely does a study discussing trauma indicate whether or not the trauma classification considered the possibility of maltreatment. Undiscovered, fabricated stories of accidents may skew the results making injuries seen from abuse appear to be consistent with those seen in accidents.

Therefore, it is important for cases of animal maltreatment to be published. With time and enough cases, patterns may emerge to allow for better assessment of when a story is consistent with the injuries seen and when it is implausible.

## 4 Conclusion

In both cases, opposing stories were provided to explain the skull fractures seen in the two kittens. A FPME was needed in each case to determine which story was more plausible. In the first case, the injuries seen were consistent with being picked up and thrown to the floor; they were inconsistent with being dropped. In the second case, both stories could potentially be plausible to explain the crushing skull fractures seen without any other injuries elsewhere on the body. Without greater details regarding the story of furniture being moved, it cannot be ruled out, nor could it be determined that the ex-girlfriend was lying.

## Data Availability

The original contributions presented in the study are included in the article/supplementary material, further inquiries can be directed to the corresponding author.
